# Effects of gene regulatory reprogramming on gene expression in human and mouse developing hearts

**DOI:** 10.1098/rstb.2012.0366

**Published:** 2013-06-19

**Authors:** Chih-Hao Hsu, Ivan Ovcharenko

**Affiliations:** Computational Biology Branch, National Center for Biotechnology Information, National Library of Medicine, National Institutes of Health (NIH), Bethesda, MD 20892USA

**Keywords:** gene regulation, lineage-specific heart enhancers, *cis*-regulatory evolution

## Abstract

Lineage-specific regulatory elements underlie adaptation of species and play a role in disease susceptibility. We compared functionally conserved and lineage-specific enhancers by cross-mapping 5042 human and 6564 mouse heart enhancers. Of these, 79 per cent are lineage-specific, lacking a functional orthologue. Heart enhancers tend to cluster and, commonly, there are multiple heart enhancers in a heart locus providing a regulatory stability to the locus. We observed little cross-clustering, however, between lineage-specific and functionally conserved heart enhancers suggesting regulatory function acquisition and development in loci previously lacking heart activity. We also identified 862 human-specific heart enhancers: 417 featuring sequence conservation with mouse (class II) and 445 with neither sequence nor function conservation (class III). Ninety-eight per cent of class III enhancers were deleted from the mouse genome, and we estimated a similar-sized enhancer gain in the human lineage. Human-specific enhancers display no detectable decrease in the negative selection pressure and are strongly associated with genes partaking in the heart regulatory programmes. The loss of a heart enhancer could be compensated by activity of a redundant heart enhancer; however, we observed redundancy in only 15 per cent of class II and III enhancer loci indicating a large-scale reprogramming of the heart regulatory programme in mammals.

## 1. Introduction

Genome-wide association studies estimate around 85 per cent of disease-causal variants residing outside protein-coding DNA [1] and large-scale international efforts to map functional elements in the human genome estimate up to 400 000 regulatory elements in the non-coding part of the human genome [2]. In contrast to genes, the regulatory elements are often lineage-specific [3] and, thus, are not amenable to the classical comparative genomics methods [4–6]. Numerous lineage-specific regulatory elements are a footprint of rapid evolutionary changes in human regulomes and represent rapid evolutionary innovation underlying the adaptive response of the human lineage. Understanding the gain, loss and evolutionary forces acting on human- (and primate-) specific regulatory elements is critical for our understanding of the gene regulatory impact on the human adaptation and disease.

As comparative genomics methods [7–9] could not be used for the identification of lineage-specific regulatory elements, we can rely only on direct enhancer (and silencer) discovery using chromatin immunoprecipitation (ChIP) with massively parallel DNA sequencing (ChIP-Seq) targeting open chromatin regions (DNaseI and similar experiments; [10,11]), bound transcription factors [12], enhancer cofactors P300 and CBP [13,14] and/or specific histone modifications [15].

In particular, ChIP-Seq experiments targeting the transcriptional co-activator P300 have been very accurate in identification of tissue-specific enhancers in the human and mouse genomes [16–19]. In this study, we compared human and mouse P300 heart enhancers sets [20]. We addressed the differences between heart enhancers conserved in mammals and heart enhancers specific to the human lineage only. We found that conserved heart enhancers have greater impact on the expression level of affected genes than lineage-specific heart enhancers. Conserved heart enhancers have higher GC-content, overlap with more CpG islands and are enriched in a known histone modification for stronger enhancers (H3k9ac) than human-specific heart enhancers. We observed a pronounced loss and gain of heart enhancers, both on the sequence and functional levels. Often, redundant/shadow enhancers prevent the loss of regulatory function upon a loss of an enhancer, while the gene expression levels display a significant change when the loss corresponds to a single heart enhancer in a locus. Our results also indicate that strong negative selection constraints active upon acquired, lineage-specific enhancers. In summary, our findings provide new insights into the lineage-specific regulatory programmes and establish a foundation for studying the regulatory diversities between species.

## 2. Results

### (a) Only a small fraction of heart enhancers is conserved between humans and mice

**Figure 1.**
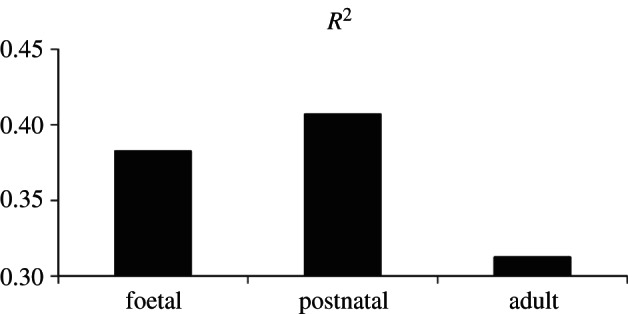
*R*^2^-value between the expression profiles of different mouse heart developmental stages with the expression profile of fetal human heart.

We compared the expression profiles of different mouse heart developmental stages with the expression profile of fetal human heart (figure 1; see §4 for details), suggesting that postnatal mouse heart has more similar expression profile as fetal human heart than foetal and adult mouse heart. Besides, Henderson *et al.* [21] have previously shown that the morphologic stage of heart development for human is over at the end of the week 7 of human embryonic development, which matches the birth in mouse. Therefore, we used P300 ChIP-Seq foetal human (gestation week 16) and postnatal mouse (day 2) heart enhancers [20]—data from the two stages that show similar developmental progression and gene expression profiles as shown in the earlier studies [20,21]. In total, 5042 human heart enhancers and 6564 mouse heart enhancers were analysed. After cross-mapping human and mouse heart enhancers [22], 1066 of them were found conserved between the two species, providing an estimate of 79 per cent of human heart enhancers being lineage-specific, whereas only a minor part of heart enhancers conserved within the mammalian branch of the evolutionary tree, consistent with previous studies [19,20].

### (b) Genomic characteristics of conserved and lineage-specific heart enhancers

**Figure 2.**
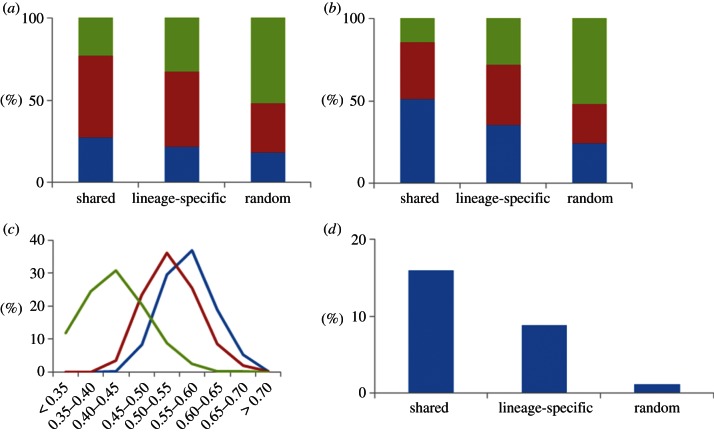
Genomic differences between shared and lineage-specific heart enhancers. (*a*) Distance to the closest TSS (green, >50 k; red, 10–50 k; blue, <10 k), (*b*) locus length (green, >500 k; red, 200–500 k; blue, <200 k), (*c*) GC-content (blue denotes shared; red, lineage-specific; green, random), and (*d*) CpG island overlaps.

To study the evolutionary trends of the heart regulatory programme, we classified human P300 heart enhancers into shared between humans and mice (dubbed simply shared for the rest of the manuscript) and lineage-specific (1066 versus 3976, respectively; figure 2). Shared heart enhancers are closer to transcriptional start sites (TSSs; Student's *t*-test *p*-value = 1.3 × 10^–18^) and populate shorter loci (Student's *t*-test *p*-value = 7.6 × 10^–34^) when compared with lineage-specific heart enhancers. In addition, shared heart enhancers feature higher GC-content (Student's *t*-test *p*-value = 2.8 × 10^–66^) and are more often located in CpG islands (Fisher's exact test *p*-value = 6.9 × 10^–11^).

### (c) Shared and lineage-specific enhancers operate in functional clusters

**Figure 3.**
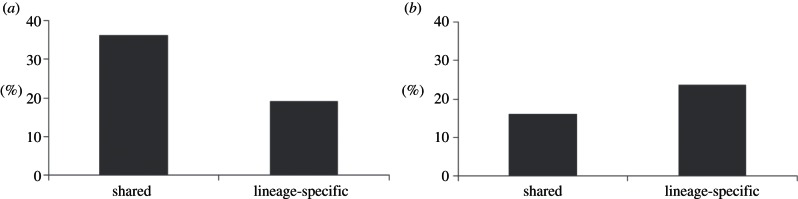
Clustering of heart enhancers with (*a*) shared and (*b*) lineage-specific heart enhancers.

Next, we performed a stochastic simulation (with 1000 replicates) to analyse the clustering between shared and lineage-specific heart enhancers. In the simulation, we randomly selected 500 shared and 500 lineage-specific heart enhancers and computed the percentage of clustered heart enhancers (more than two heart enhancers located in the same locus). Almost twice as many shared heart enhancers are clustered with shared heart enhancers (36%) than with lineage-specific heart enhancers (19%; figure 3*a*; Fisher's exact test *p*-value = 1.8 × 10^–9^). More lineage-specific heart enhancers are clustered with lineage-specific heart enhancers (24%) than shared heart enhancers (16%) as well (figure 3*b*; Fisher's exact test *p*-value = 0.002). This suggests the importance of regulatory redundancy in loci of genes expressed in the heart. Also, the reduced clustering of shared and lineage-specific enhancers indicates the separation of ancestral and novel regulatory programmes with recent regulatory structures targeting and building up within loci that previously lacked heart expression.

### (d) Three categories of lineage-specific enhancers

To study how loss or gain of heart enhancers affects the expression of flanking genes and their function in the heart, we further partitioned lineage-specific human heart enhancers into three classes based on evolutionary sequence conservation and the strength of ChIP-Seq signal in their mouse sequence orthologues. In particular, we were concerned with possible false positive lineage-specific elements associated with strict ChIP-Seq cut-offs used to call a region an enhancer. To avoid a potential negative impact of experimental uncertainties, we defined class I representing equivocal lineage-specific enhancers (3114 regions) whose sequences were conserved in the mouse genome and whose mouse homologous regions did not overlap ChIP-Seq peaks, but featured ChIP-Seq read count exceeding the genome-wide background level (see §4 for details). By definition, class I enhancers represent a mix of true human-specific heart enhancers and enhancers potentially shared in humans and mice that were not assigned to the shared class due simply to the low signal in mouse experiment. Therefore, all conclusions stemming from the analysis of the class I data should be taken with a grain of salt. The remaining true lineage-specific human heart enhancers (for which we were confident no significant mouse heart enhancer functionality could be detected) were split into the class II (417 regions) featuring sequence conserved with mouse and class III (445 regions) featuring no sequence homology in the mouse genome. To prevent indiscriminative results, class I enhancers, which could represent weak heart enhancers in mouse and, thus, be reclassified as shared enhancers, were mainly excluded from the following functional studies.

### (e) Majority of non-conserved human-specific heart enhancers were lost in the mouse lineage

There are two evolutionary possibilities for giving rise to a human-specific enhancer with no sequence homology in the mouse genome (class III): either a sequence insertion in the human lineage or sequence deletion in the mouse lineage. To delineate between these two scenarios, we used sequence alignments between human and seven distant species forming an out-group (dog, cat, horse, cow, opossum, chicken and frog), to study the evolutionary history of the genomic regions in question. Ninety-eight per cent (436/445) of these enhancer sequences are present in at least one of the seven distant species. This points to the ancestral nature of class III enhancers and supports a model of enhancer sequence deletion in the mouse lineage for the majority of enhancers forming this class. This, in turn, indicates that the reshaping of the regulatory genome is largely a result on enhancer sequence loss. As there are no fewer mouse heart enhancers than human heart enhancers (see §2*a* for raw counts), this enhancer loss should have been compensated by enhancer gain. Class II (and partially class I) enhancers can possibly shed the light on the enhancer gain path. Novel human heart enhancers should have been formed by heart enhancer function acquisition without sequence loss in mouse (class II) or gradual function gain with or without sequence divergence (class I).

### (f) Highly expressed heart genes rely on functionally conserved enhancers

**Figure 4.**
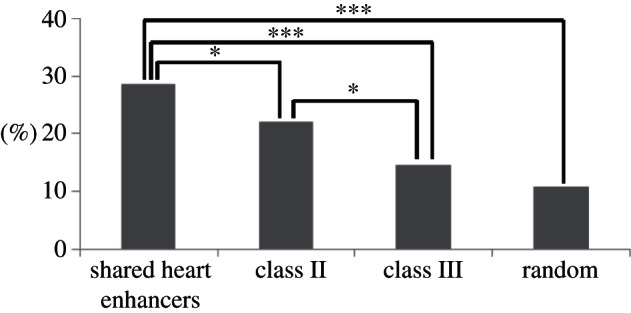
Percentage of heart enhancers and controls located in the loci of genes highly expressed in the heart. Asterisks indicate statistical significance (*p-value < 0.05 and ***p-value < 0.001).

To study how different heart enhancers affect the expression of flanking genes, we identified 1000 highly expressed heart genes (see §4 for details) and calculated the percentage of heart enhancers located in the loci of these highly expressed heart genes (figure 4). Significantly more shared heart enhancers are located in the loci of highly expressed heart genes than either class II/III human-specific heart enhancers (Fisher's exact test *p*-value = 0.045 and 1.9 × 10^–5^, respectively) or random expectation (5000 randomly selected regions; Fisher's exact test *p*-value = 1.7 × 10^–21^). This suggests elevated levels of negative selection acting on heart enhancers located proximally to genes highly expressed in the heart, both from the sequence and function selection viewpoint. In addition, more conserved human-specific heart enhancers (class II) are located in the loci of highly expressed heart genes than human-specific heart enhancers lacking sequence similarity with mouse (class III; Fisher's exact test *p*-value = 0.034). As class III corresponds to sequences that have been predominantly lost in the mouse lineage (see §2*e*), these results demonstrate that the decreased strength of selection acting on the class III elements and allowing their loss in the mouse lineage was probably the result of the reduced importance of the class III elements in the developmental heart programme, as depicted through their genomic location away from genes highly expressed in the heart.

### (g) Shared heart enhancers are associated with strong enhancers

**Figure 5.**
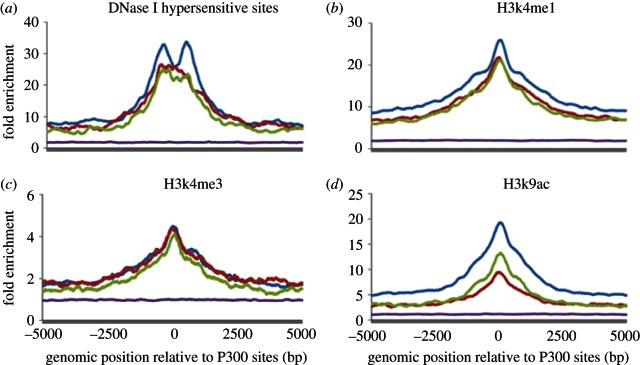
(*a*–*d*) Distribution of histone modifications around heart enhancers. Heart enhancers are contrasted to a random set of 1000 non-coding-conserved sequences. Blue denotes shared; red, class II; green, class III; purple, random.

Histone modifications have been shown to play a critical role in determining spatio-temporal gene expression patterns in vertebrate genomes [15,23] and several histone modifications have been established as reliable indicators of gene regulatory elements. For this project, we were particularly interested in H3K4me1 and H3K9ac histone modifications associated with enhancers and H3K4me3 associated with promoters [24,25]. Besides, we also used DNase I hypersensitive sites to study the chromatin accessibility [10]. We analysed the distribution of distinct histone modifications around heart enhancers that belong to different categories (figure 5). Among all histone modifications, H3K9ac shows the most significant difference between shared heart enhancers and class II/III human-specific heart enhancers (Fisher's exact test *p*-value = 2.1 × 10^–17^ and 1.2 × 10^–7^, respectively), with shared enhancers demonstrating significantly stronger association with H3K9ac. As H3K9ac is known to be characteristic of strong, distant enhancers [25], it is likely that shared enhancers are more potent in activating gene expression. In addition, elevated DNase I levels in proximity to shared heart enhancers indicate higher levels of chromatic accessibility to *trans*-acting factors in the genomic regions they occupy (figure 5).

### (h) Human heart enhancers are under negative selection pressure

We used two methods—derived allele frequency (DAF) and McDonald–Kreitman test (MK test)—to investigate selective constraints, under which heart enhancers evolve. We downloaded human variation data generated by the 1000 Genomes Project [26] and used the ancestral allele information based on six-way primate alignment from the Ensembl compara database [27] to determine the DAF for each variant. In addition, we used 16 315 pseudogenes from the Pseudogenes.org database [28] as the neutral reference. All of shared heart enhancers and class II/III human-specific heart enhancers showed a higher fraction of low-frequency variants (DAF ≤ 5%) compared with the neutral reference (table 1; Fisher's exact test *p*-value = 6.8 × 10^–52^, 3.1 × 10^–51^ and 7.4 × 10^–29^, respectively), suggesting all classes of human heart enhancers are under negative selection pressure.

**Table 1.** DAF in heart enhancers versus controls (pseudogenes).

	shared heart enhancers	class II	class III	pseudogenes
DAF ≤ 5%	10 640 (74.3%)	4793 (77.1%)	5006 (74.7%)	192 359 (68.4%)
total	14 311	6213	6701	281 111
*p*-value	6.8×10^–52^	3.1×10^–51^	7.4×10^–29^	

We also used MK test to study the selection over human heart enhancers. Human polymorphism sites (*P*; variation within species) were contrasted to the non-polymorphic human sites different from their chimpanzee (*Pan troglodytes*) counterparts (*D*; fixed differences or variation between species with no inter-species variation). *P* and *D* enhancer counts (*P*_e_ and *D*_e_, respectively) were compared with the corresponding neutral reference counts *P*_n_ and *D*_n_ calculated using pseudogenes. Neutrality index defined as (*P*_e_/*D*_e_)/(*P*_n_/*D*_n_) was determined for each type of heart enhancers. All of three types of heart enhancers were subject to negative selection (neutrality index > 1; table 2), where class II human-specific heart enhancers have the largest neutrality index, which is consistent with the DAF result (table 1). These results are particularly interesting for the class II and III heart enhancers, as they indicate no decrease in selective pressure on novel heart enhancers (class II) or heart enhancers prone to loss in other lineages (class III).

**Table 2.** Neutrality test (MK test) for distinct heart enhancers.

	polymorphism (*p*)	fixed difference (*D*)	neutrality index	*p*-value
shared heart enhancers	12 893	10 148	1.61	2.1×10^–274^
class II	5736	4312	1.68	5.8×10^–147^
class III	6283	5400	1.47	3.5×10^–95^
pseudogenes	349 789	442 395	1.00	

### (i) Biological function of heart genes flanking shared heart enhancers

**Figure 6.**
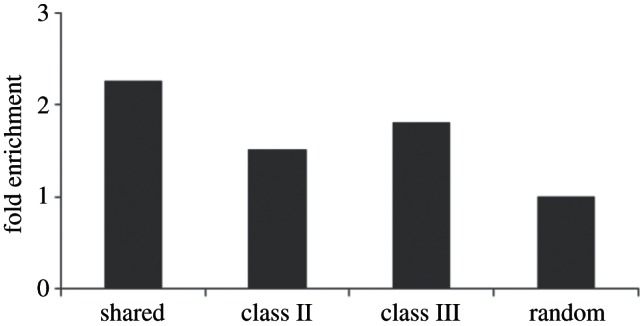
Cardiac GO-term enrichment in genes flanking heart enhancers. Heart enhancers are contrasted to a random set of 1000 non-coding-conserved sequences.

We performed a gene ontology (GO) analysis to quantify the association of heart enhancers with the biological function of flanking genes. We selected all cardiac GO terms containing ‘heart’, ‘cardiac’ or ‘cardio’ in their names for the analysis. Totally, 398 cardiac GO terms and 346 genes annotated to these categories were obtained. All categories of heart enhancers display enrichment in cardiac GO terms confirming the heart function of genes flanking these enhancers (figure 6). When different enhancer categories are compared, shared heart enhancers display the strongest enrichment in the loci of heart genes (Binomial test *p*-value = 9.4 × 10^–10^), again alluding to the important function this category of heart enhancers plays in heart development. Notably, the least-fold enrichment was observed for the class II elements indicating that the gain of regulatory function takes place in loci previously not strongly involved into the heart regulatory programme. At the same time, the class III fold enrichment almost reaches the level of shared enhancers suggesting that the loss of the class III enhancer counterparts in the mouse genome had a direct impact on the mouse heart regulatory programme.

### (j) Loss of heart enhancers in the mouse lineage

There are no functional orthologues of class II and class III human-specific heart enhancers in the mouse genome, which allows us to directly quantify the effects of an enhancer loss on the level of gene expression between human and mouse. To analyse whether the lack of heart enhancer function in the corresponding loci of the mouse genome had a footprint on the expression of flanking genes, we investigated whether the loss of a heart enhancer in the mouse genome (or an enhancer gain in the human genome) was balanced by a gain of a new heart enhancer in the same mouse locus and/or the effects of the enhancer loss/gain were mitigated by other redundant/shadow enhancers in that locus. We centred our analysis on the human loci that have orthologous counterparts in the mouse genome. Within the mouse locus counterparts, 3814 mouse heart enhancers were mapped and 2748 of them were mouse-specific (based on function conservation, not necessarily sequence conservation). In addition, because human-specific heart enhancers can reside in the same locus with shared heart enhancers that are sufficient to maintain the heart regulatory activity in the locus (figure 3), we used only singleton heart enhancers (heart enhancers without any other heart enhancers located in the same locus) for this analysis. In total, 220 singleton shared heart enhancers, 101 singleton class II heart enhancers and 119 singleton class III heart enhancers were used.

**Figure 7.**
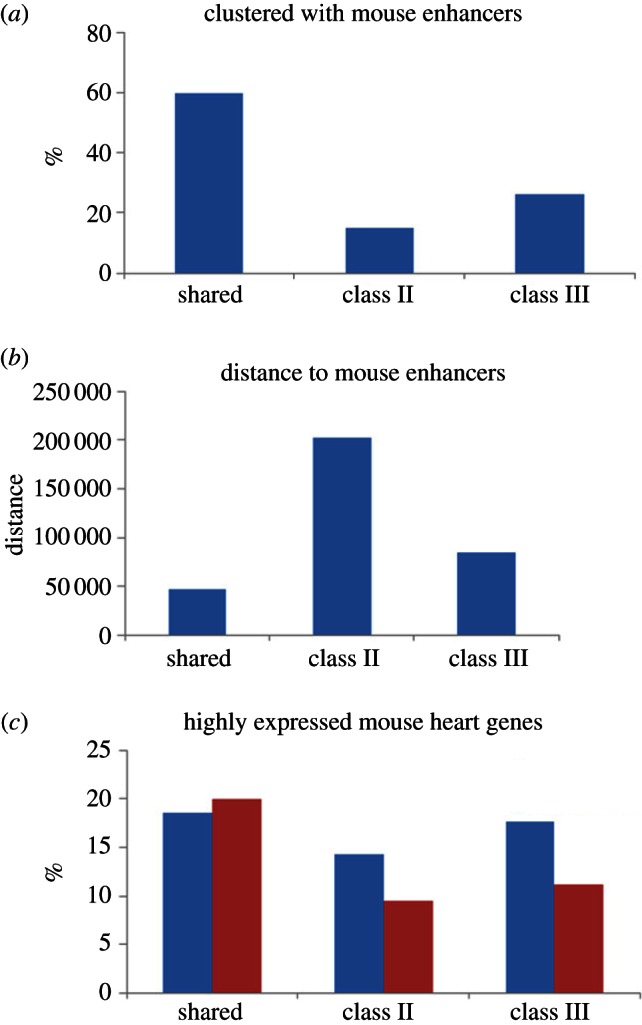
Clustering of the syntenic mouse loci to singleton human heart enhancers with mouse-specific enhancers. The syntenic mouse loci did not host a single heart enhancer for the class II and class III heart enhancers. (*a*) Percentage of three distinct human heart enhancers whose loci also contains at least one mouse-specific heart enhancers in the mouse genome. (*b*) Distance between human heart enhancers and mouse-specific heart enhancers when they are clustered in the same loci. (*c*) The fraction of highly expressed genes flanking mouse heart enhancers (blue denotes clusters; red, non-clusters).

In contrast to the 60 per cent shared heart enhancers that contain at least one other mouse-specific heart enhancer in the orthologous mouse locus, only 15 per cent of class II and 26 per cent of class III enhancers feature the same trend (figure 7*a*). As the class III almost exclusively corresponds to the loss of enhancers in mouse genome (as opposed to an enhancer gain in the primate lineage), these results suggest that a loss of a heart enhancer in mouse is not balanced by an independent heart enhancer gain in the host mouse locus. As singleton heart enhancers represent only a minority of all heart enhancers, the observed heart regulatory activity eradication in many loci with redundant enhancers can be compensated by activity of other heart enhancers in case of a single enhancer loss. However, these results identify single enhancer loci open to regulatory reprogramming, and indicate that the heart activity has been lost or gained in many of them. In addition, even though the mouse orthologous loci of 15 per cent of class II heart enhancers feature additional mouse-specific heart enhancers, the average distance between the mouse-specific heart enhancer and the mouse orthologue of the human-specific heart enhancers is over 200 kb and is fourfold larger than the distance between the mouse orthologues of shared human heart enhancers and their neighbouring mouse-specific heart enhancers (figure 7*b*). As class II represents the primary set for heart enhancers that have been gained in the human lineage, this suggests that this is a case of convergent evolution with independent heart enhancer acquisition in human and mouse genomes might still differ in the regulatory mechanisms associated with the acquired heart regulatory activity.

The ultimate impact of the regulatory change following the loss and gain of heart enhancers is the change in the heart expression levels of genes flanking the affected enhancers. In particular, we were interested in learning if (i) the loss of a human enhancer in the mouse lineage leads to a decreased level of heart expression and (ii) the presence of another mouse-specific enhancer in proximity to the site of loss could mitigate the impact on the expression level. To validate this observation, we studied the expression level of genes flanking these singleton human-specific heart enhancers in the mouse genome. Indeed, we observed a significant decrease in the fraction of highly expressed mouse heart genes flanking non-clustered (those that do not have an additional mouse enhancer in the locus) mouse counterparts of class II (binomial test *p*-value = 0.03) and class III (binomial test *p*-value = 0.09) human enhancers when compared with mouse orthologues of shared enhancers (figure 7*c*). The presence of another mouse enhancer in the locus (clustered) mitigates the impact on the gene expression level upon a loss of a heart enhancer (figure 7*c*), but the qualitative observations did not reach statistical significance in our analysis owing to the low fraction of class II and class III mouse counterparts featuring another mouse heart enhancer in the locus (figure 7*a*). These results demonstrate that the loss and gain of singleton heart enhancers in the locus has a pronounced impact on the regulatory activity of the corresponding genes.

## 3. Discussion

In this study, we identified 1066 heart enhancers shared by humans and mice, and 3976 lineage-specific human heart enhancers. By comparing the distribution of distinct genome features between shared and lineage-specific heart enhancers in the human genome, we found that lineage-specific heart enhancers are more distant to transcription start sites and are located in longer loci than shared enhancers suggesting that even though plenty of distal regulatory elements are discovered in the vertebrate genomes [29,30], the most important regulatory elements are located closer to the affected genes [31] and proximal regulatory elements have higher impact to the affected genes than distal regulatory elements [32]. In addition, we found that shared heart enhancers have higher GC-content and overlap with more CpG islands, which is consistent with results showing that functional conserved elements always have higher GC-content and overlap with CpG islands [33].

Clustering analysis of shared and lineage-specific human heart enhancers as shown in figure 3 indicates that lineage-specific human heart enhancers tend to cluster with lineage-specific human heart enhancers more than shared human heart enhancers. Furthermore, we found that sequence orthologues of human-specific heart enhancers are rarely clustered with mouse-specific heart enhancers indicating loss of regulatory function in the mouse genome with no functional compensation by redundant enhancers in the majority of the cases. Expression analysis for the flanking genes between shared and human-specific heart enhancers shows that shared heart enhancers have a greater impact on the expression level of affected genes than human-specific heart enhancers. A functional footprint study also shows that enrichment of the H3k9ac mark associated with strong enhancer is almost twofold greater in shared heart enhancers than human-specific heart enhancers, suggesting that the enhancer activity can be higher in shared heart enhancers than human-specific heart enhancers. GO analysis also indicates that more genes affected by shared heart enhancers are related to heart function than human-specific heart enhancers and no particular cardiac GO term was only enriched in the human-specific heart enhancers. All of these results suggest that even though human-specific heart enhancers are lost in the mouse genome and no counterparts exist in the mouse genome to recover the affected genes, these affected genes are not as important to the heart functions as those affected by shared heart enhancers.

While the DNA sequences of non-conserved human-specific heart enhancers were predominantly deleted in the mouse genome, investigation of selective constrains indicates that these sequences are still evolving under negative selection pressure in humans. In addition, we found that genes affected by non-conserved human-specific heart enhancers are more related to heart functions than conserved human-specific heart enhancers based on GO analysis and more counterparts in the mouse genome are in the same loci as the non-conserved human-specific heart enhancers than conserved human-specific heart enhancers, suggesting that non-conserved human-specific heart enhancers still play an important role in heart function.

In summary, this study provides new insights into the evolution and functional role of lineage-specific regulatory elements in mammals.

## 4. Material and methods

### (a) Comparison between different mouse heart developmental stages with fetal human heart

Expression profiles of three different mouse heart developmental stages, i.e. foetal (E11.5; GSE1479), postnatal (one week; GSE38754) and adult (nine months; GSE41810), were compared with the expression profile of fetal human heart (GSE1789). Expression profile of human genes were mapped into the mouse genome using Homologene (http://www.ncbi.nlm.nih.gov/homologene) and *R*^2^-value was calculated for each expression profile of different mouse heart developmental stage to study the correlation of the expression profiles between foetal human heart and three different mouse heart developmental stages.

### (b) Shared and lineage-specific heart enhancers

P300 ChIP-Seq human heart enhancers (gestational week 16; 5042 sequences) and mouse heart enhancers (postnatal day 2; 6564 sequences) were downloaded from May *et al.* [20]. Mouse heart enhancers were mapped into the human genome using the liftOver tool [22] and resulted in 6230 orthologous human sequences. Overlapping human heart enhancers and orthologous sequences of mouse heart enhancers were termed shared heart enhancers (1066 sequences). Other human heart enhancers were treated as lineage-specific heart enhancers (3976 sequences).

### (c) Locus length

**Figure 8.**
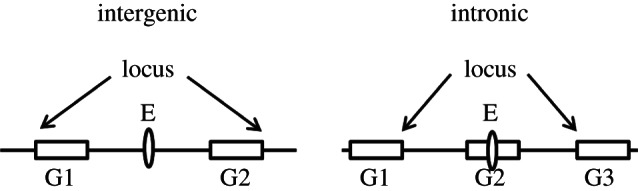
Determination of locus length for intergenic/intronic heart enhancers.

Locus length for intergenic/intronic heart enhancers was determined separately (figure 8). For an intergenic heart enhancer, the locus encompasses two flanking genes and the intergenic region separating them; for an intronic heart enhancer, the locus comprises the host gene and the two intergenic intervals flanking it.

### (d) Lineage-specific heart enhancers were partitioned into three classes

All lineage-specific human heart enhancers were mapped into the mouse genome using liftover [22] and resulted in 3531 orthologous mouse sequences. After that, lineage-specific heart enhancers were separated into three classes based on their orthologous sequences in the mouse genome. Class III consisted of 445 lineage-specific heart enhancers that cannot be mapped into the mouse genome—non-conserved, human-specific heart enhancers. Out of 3531 orthologous sequences in the mouse genome, 3114 had higher ChIP-Seq read densities compared with the average ChIP-Seq read densities for all mouse-human evolutionarily conserved regions (ECRs; [34]) and were considered as equivocal heart enhancers (class I). Other 417 orthologous sequences whose ChIP-Seq read densities did not exceed the average ChIP-Seq read densities for all ECRs were considered conserved human-specific heart enhancers (class II).

### (e) Top 1000 highly expressed human and mouse heart genes

Gene expression data in foetal human heart were downloaded from the GEO database (GSE1789), and average expression levels across five normal foetal heart samples were calculated. In addition, gene expression data for 10 human non-heart tissues in the HG-U133_Plus_2.tissue-mixture-data-set were downloaded from Affymetrix (http://www.affymetrix.com) and average expression levels for these 10 human non-heart tissues were calculated. For both of these two expression datasets, a small arbitrary number (16) was added to each expression value to avoid misleading readings for low expression values. Log-transformation was performed for the expression level of each gene, and ratio of expression level in the heart tissue to that in the non-heart tissue was calculated. The top 1000 genes with the highest ratio were selected as the highly expressed human heart genes.

For the mouse genome, gene expression data for one-week-old mouse in the heart tissue were downloaded from the GEO database (GSE38754), and average gene expression levels across five samples were calculated. Besides, gene expression data for 90 mouse non-heart tissues were downloaded from the GEO database (GSE10246). Normalization similar to the human genome was performed to get the top 1000 highly expressed mouse heart genes.

### (f) Estimation of selection pressure using 1000 genome project data

SNP data generated by the 1000 Genomes Project [26] were used to study selective constrains for distinct classes of heart enhancers. Two methods (DAF and MK test) were used to estimate selection pressure. To determine DAF for each SNP, we downloaded the ancestral allele information based on a six-way primate alignment from the Ensembl compara database [27]. SNP sites for each class of heart enhancers were determined and DAF for these SNP sites were counted. To analyse the selective pressure on human heart enhancers, we used 5 per cent as the cut-off threshold for DAF because variants with DAF > 5% are defined as common variants and SNPs with DAF ≤ 5% are often referred to as low-frequency variants [35]. Fisher's exact test was used to determine the significance of DAF for each class of heart enhancers compared with the neutral reference (pseudogenes).

The MK test was also used to estimate selective constrains over human heart enhancers. The MK test compares the difference between polymorphism (*P*), i.e. variation within species and fixed difference (*D*), i.e. variation between species but not within species, and studies the fixed rate of variants. Polymorphism (*P*) was estimated by the sites of SNP across heart enhancers and fixed difference (*D*) was determined by the difference between the number of nucleotide differences between human and chimpanzee (*d*) and the heterozygous sites across heart enhancers (**π**), i.e. *D* = *d*−**π**. The ratio between polymorphism and fixed difference was calculated for each class of heart enhancers (*P*_e_/*D*_e_) and was compared with the ratio of the neutral reference (*P_n_*/*D_n_*). The Neutrality index is defined as (*P*_e_/*D*_e_)/(*P*_n_/*D*_n_) and if the neutrality index is greater than 1 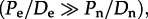 this indicates that human heart enhancers have been subject to negative selection. Otherwise, if the neutrality index is less than 1 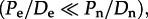 human heart enhancers have been subject to positive selection. Fisher's exact test was also used to estimate the significance of MK-test.

### (g) Gene ontology analysis

To determine whether distinct classes of heart enhancers are overrepresented near some particular classes of heart genes, the closest genes for each class of heart enhancers were used as the test dataset (*T*), and all genes near all heart enhancers were treated as the background dataset (*B*). Fisher's exact test was performed to calculate the *p*-value for each GO term and the Bonferroni multiple-testing correction was used to determine whether a GO term is significantly enriched in the test dataset. In addition, to determine whether distinct classes of heart enhancers are enriched in different heart genes and are associated with different heart function, only GO terms containing ‘heart’, ‘cardiac’ or ‘cardio’ in their names and genes associated with these GO terms were used in our analysis. Totally, 398 GO terms and 346 genes were used.

### (h) Clustering of human-specific heart enhancers and mouse-specific heart enhancers

To study whether human-specific heart enhancers are balanced by mouse-specific heart enhancers so that even though human-specific heart enhancers were lost in the mouse genome their flanking genes are still activated by the compensated mouse-specific heart enhancers, first, we identified singleton human heart enhancers, which were not clustered with any other human heart enhancers in the same loci, for each class of human heart enhancers. In total, 220 shared heart enhancers, 101 conserved human-specific heart enhancers (class II) and 119 non-conserved human-specific heart enhancers (class III) were singleton human heart enhancers. After that, all human-specific heart enhancers were mapped into the mouse genome using liftOver [22] and the percentage of singleton human heart enhancers whose orthologous loci in the mouse genome contained at least one mouse-specific heart enhancers was calculated for each class of human heart enhancers.
